# Supplementary material for: Poleward migration of western North Pacific tropical cyclones related to changes in cyclone seasonality
Source: Nat Commun. 2021 Oct 27;12:6210. doi: 10.1038/s41467-021-26369-7 (PMC8551271; doi:10.1038/s41467-021-26369-7)
Supplement: Supplementary file 1 — Supplementary Information [file 41467_2021_26369_MOESM1_ESM.pdf]

Supplementary Information

Poleward migration of western North Pacific tropical cyclones related to changes in cyclone seasonality

Xiangbo Feng[1*], Nicholas P. Klingaman[1], Kevin I. Hodges[1]

[1] National Centre for Atmospheric Science and Department of Meteorology, University of Reading, Reading, United Kingdom

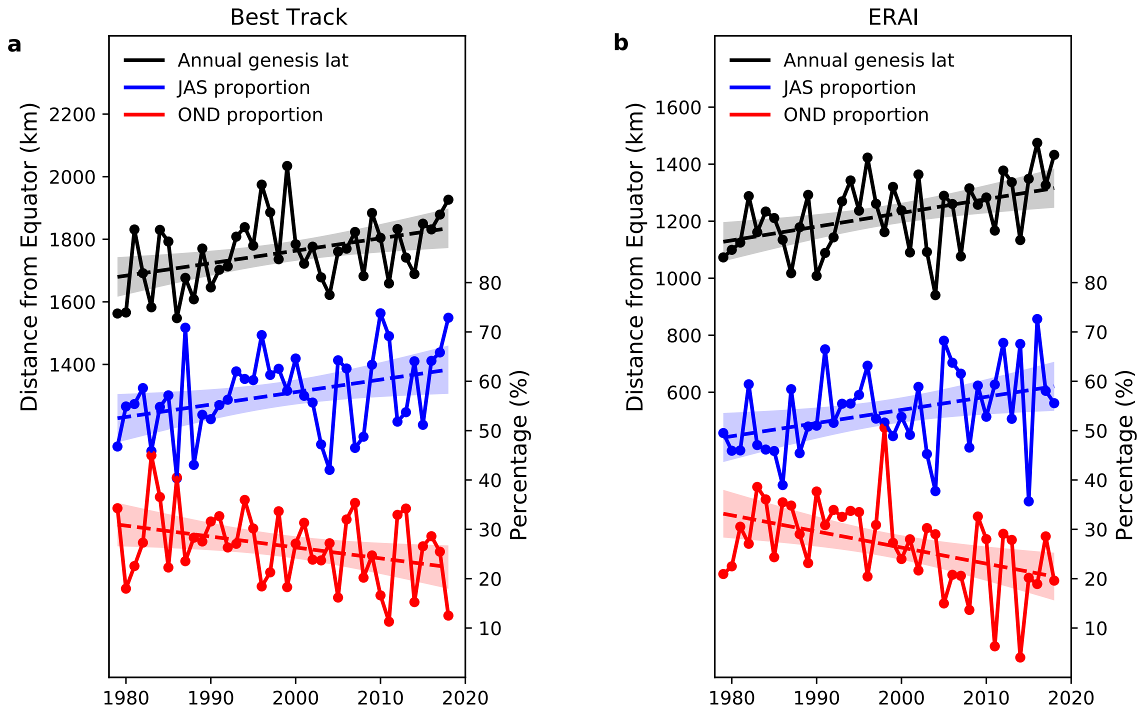

Supplementary Figure 1: Changes in annual-mean latitude and seasonal relative frequency of tropical cyclone genesis.

(a) Timeseries of annual-mean latitude of TC genesis (black), and timeseries of peak-season (July–September; JAS, blue) and late-season (October–December; OND, red) relative frequencies of TC genesis in the year, over 1979–2018, in the Best Track ensemble mean. (b) as (a), but in the ERA-Interim TC dataset. Dashed lines represent the linear trend, with shading showing 95% confidence interval for the linear fit; the El Niño Southern Oscillation and Pacific Decadal Oscillation effects are removed by a multivariate regression; latitude is converted to distance from the equator (km).

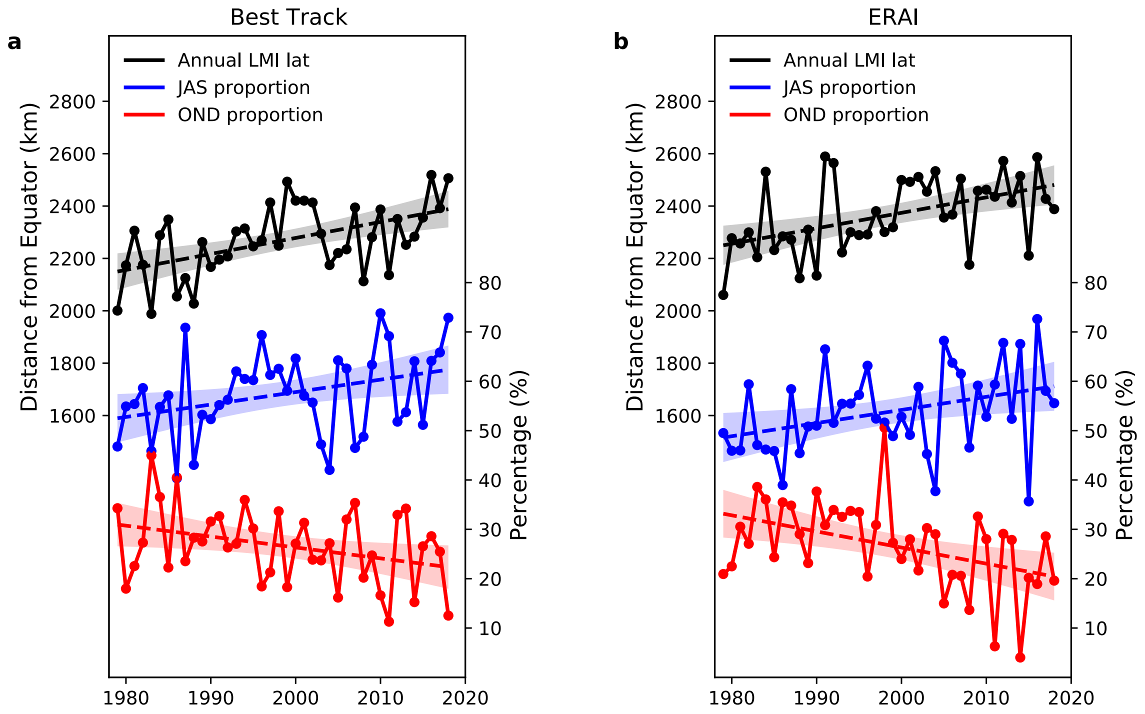

Supplementary Figure 2: Changes in annual-mean latitude and seasonal relative frequency of tropical cyclone lifetime-maximum intensity.

As Supplementary Figure 1, but for TC lifetime-maximum intensity (LMI).

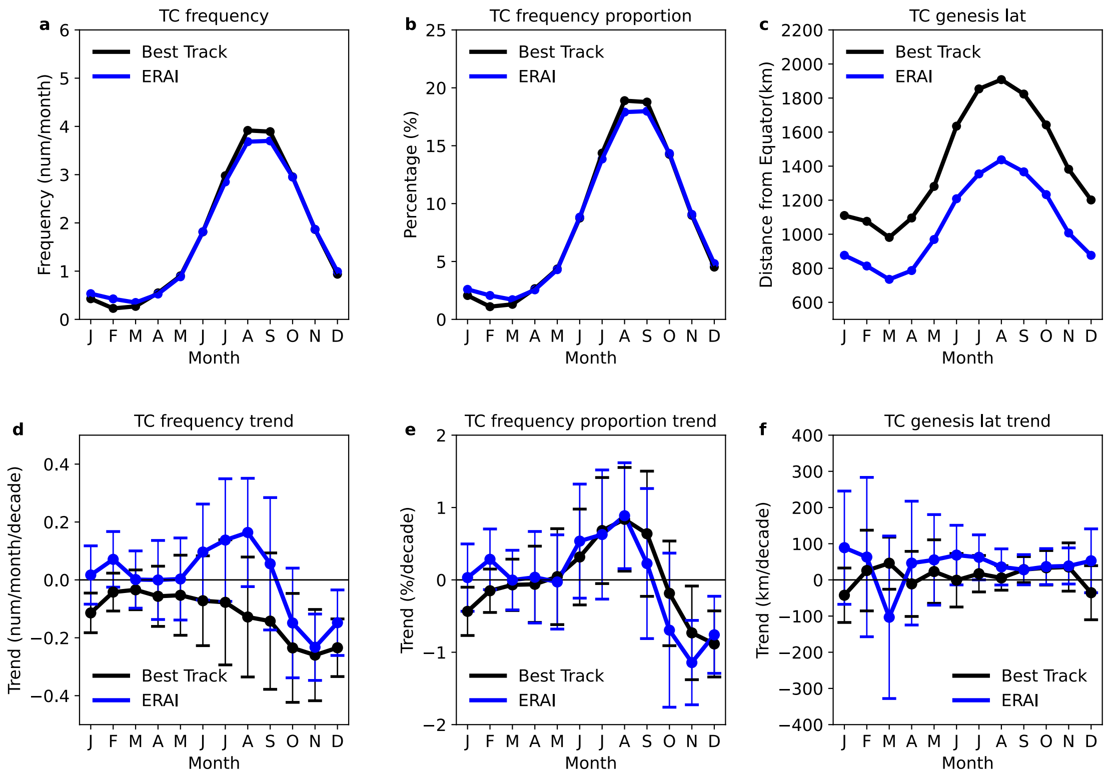

Supplementary Figure 3: Seasonality of tropical cyclone genesis, and changes in seasonality. (a-c): Climatology of 3-month rolling averages of monthly frequency, monthly relative frequency and monthly latitude for TC genesis, over 1979–2018, in the Best Track ensemble mean (black) and ERA-Interim (blue) TC datasets. (d-f): Linear trends in 3-month rolling averages of monthly frequency, monthly relative frequency and monthly latitude for TC genesis, over 1979–2018, in the Best Track ensemble mean (black) and ERA-Interim (blue) TC datasets. In (c) and (f), latitude is converted to distance from the equator (km); in the lower panels, the error bars show 95% confidence interval for the trends; the El Niño Southern Oscillation and Pacific Decadal Oscillation effects are removed by a multivariate regression.

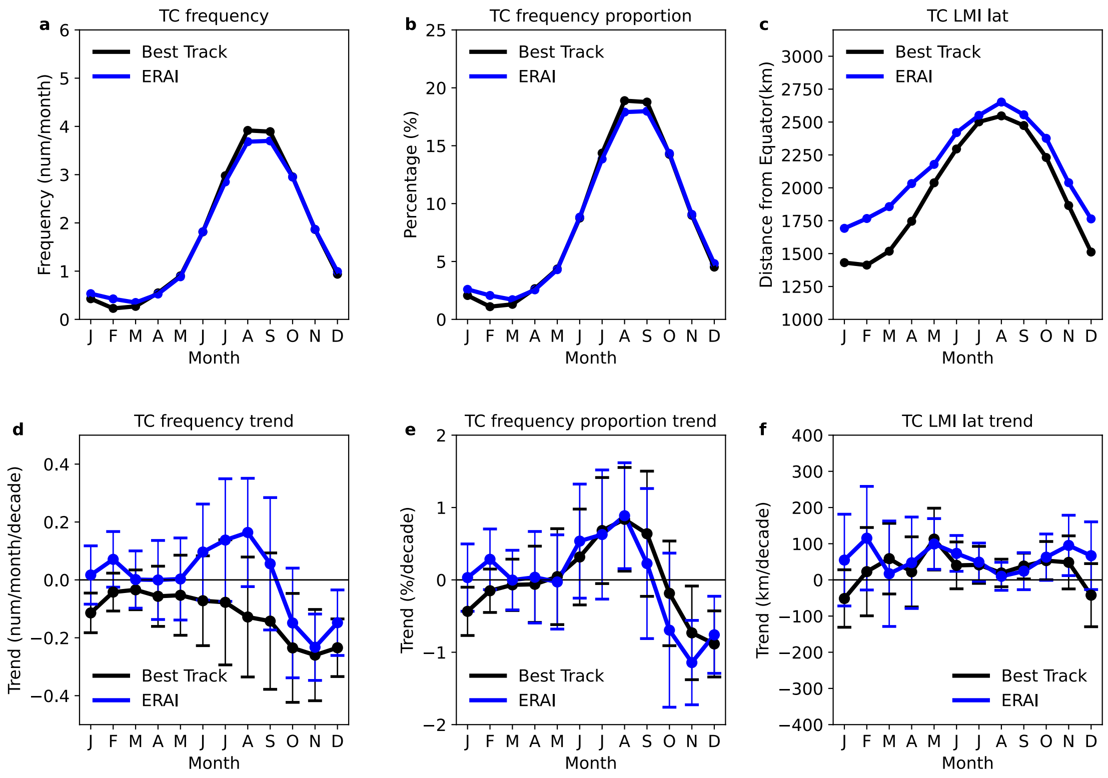

Supplementary Figure 4: Seasonality of tropical cyclone lifetime-maximum intensity, and changes in seasonality.

As Supplementary Figure 3, but for TC lifetime-maximum intensity (LMI).

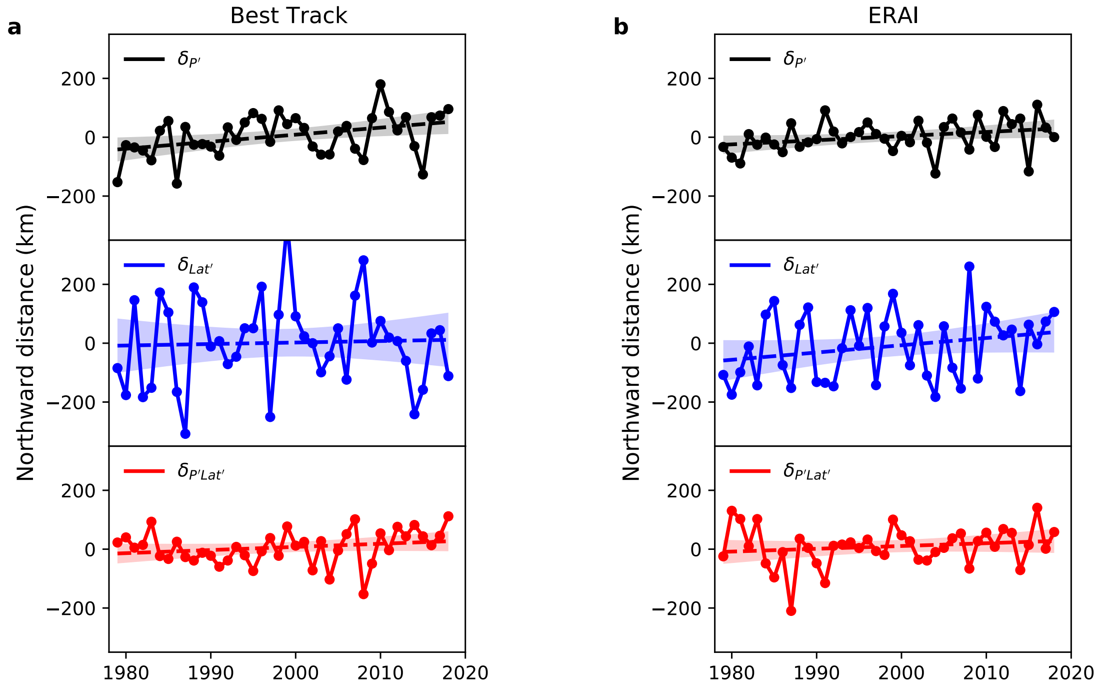

Supplementary Figure 5: Changes in three seasonality components of annual-mean latitude of tropical cyclone genesis.

(a): Decompositions of annual-mean latitude of TC genesis by three seasonality contributing components, representing the effects of departures (from the respective monthly means) of monthly relative frequency ($\delta_{P'}$, black), monthly latitude ($\delta_{Lat'}$, blue) and the covariance of the two terms ($\delta_{P'Lat'}$, red), respectively, over 1979–2018, in the Best Track ensemble mean. (b) as (a), but in the ERA-Interim TC dataset. Dashed lines represent the linear trend, with shading showing 95% confidence interval for the linear fit; the El Niño Southern Oscillation and Pacific Decadal Oscillation effects are removed by a multivariate regression; latitude is converted to distance from the equator (km).

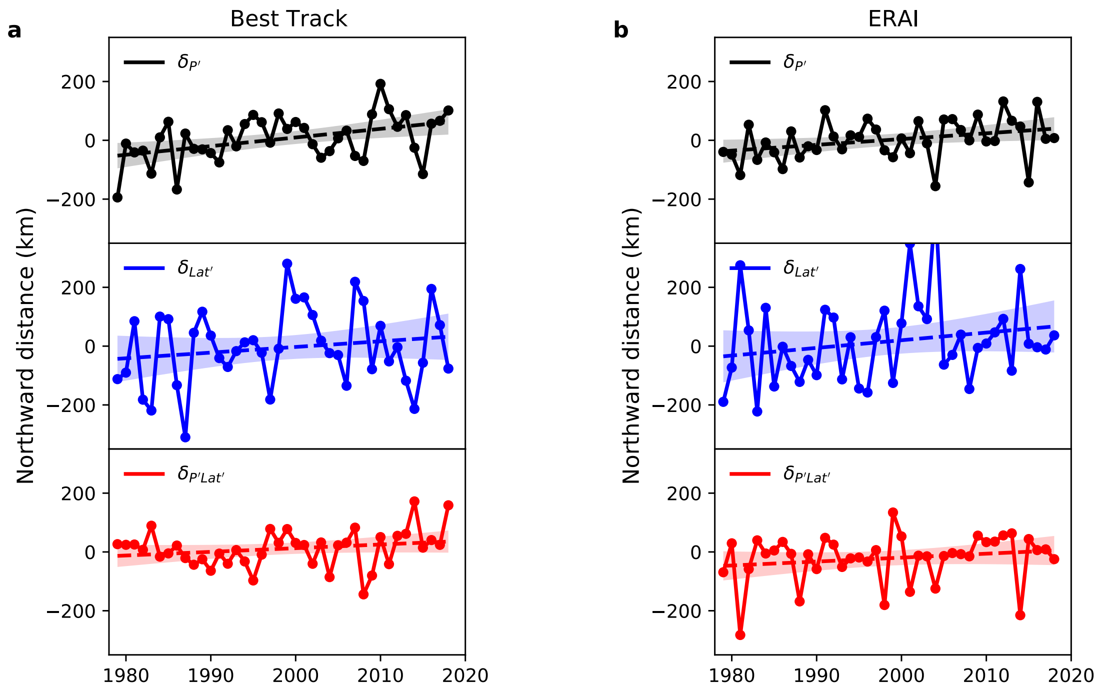

Supplementary Figure 6: Changes in three seasonality components of annual-mean latitude of tropical cyclone lifetime-maximum intensity.

As Supplementary Figure 5, but for TC lifetime-maximum intensity (LMI).

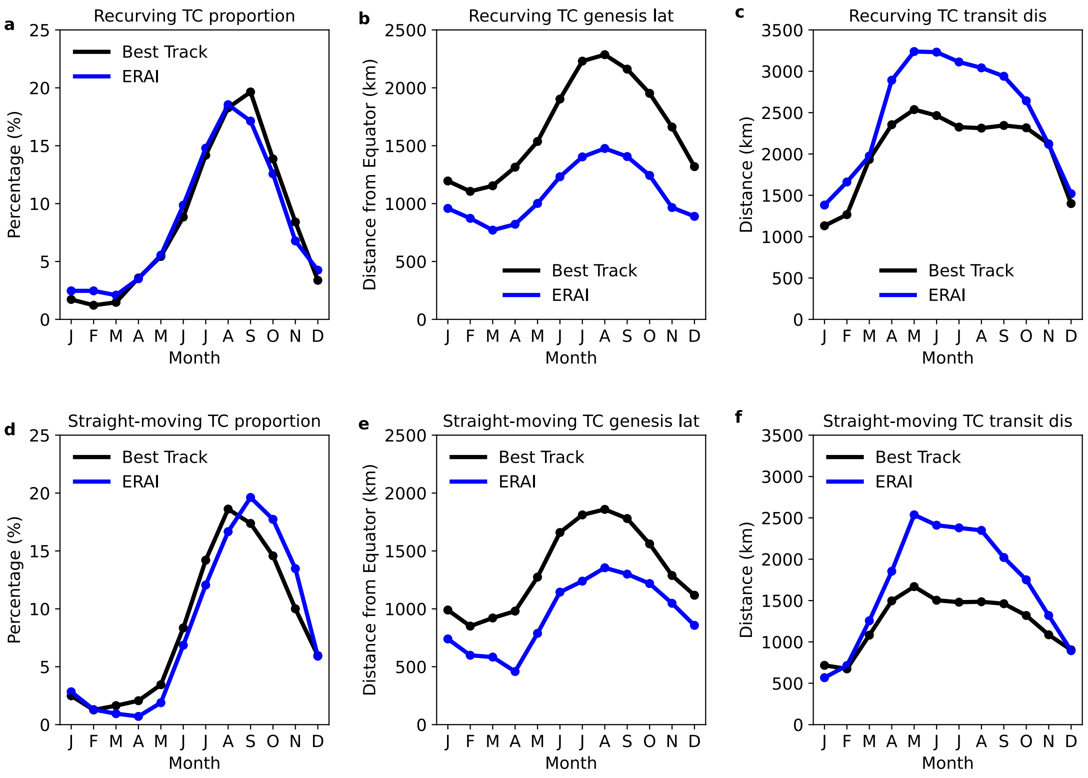

Supplementary Figure 7: Seasonality of genesis for recurving and straight-moving tropical cyclones.

(a-c): Climatology of 3-month rolling averages of monthly relative frequency, monthly genesis latitude and monthly net latitudinal transit distance for recurving TCs, over 1979–2018, in the Best Track ensemble mean (black) and ERA-Interim (blue) TC datasets. (d-f), as (a-c), but for straight-moving TCs. In (b-c) and (e-f), latitude and latitudinal distance are converted to km.

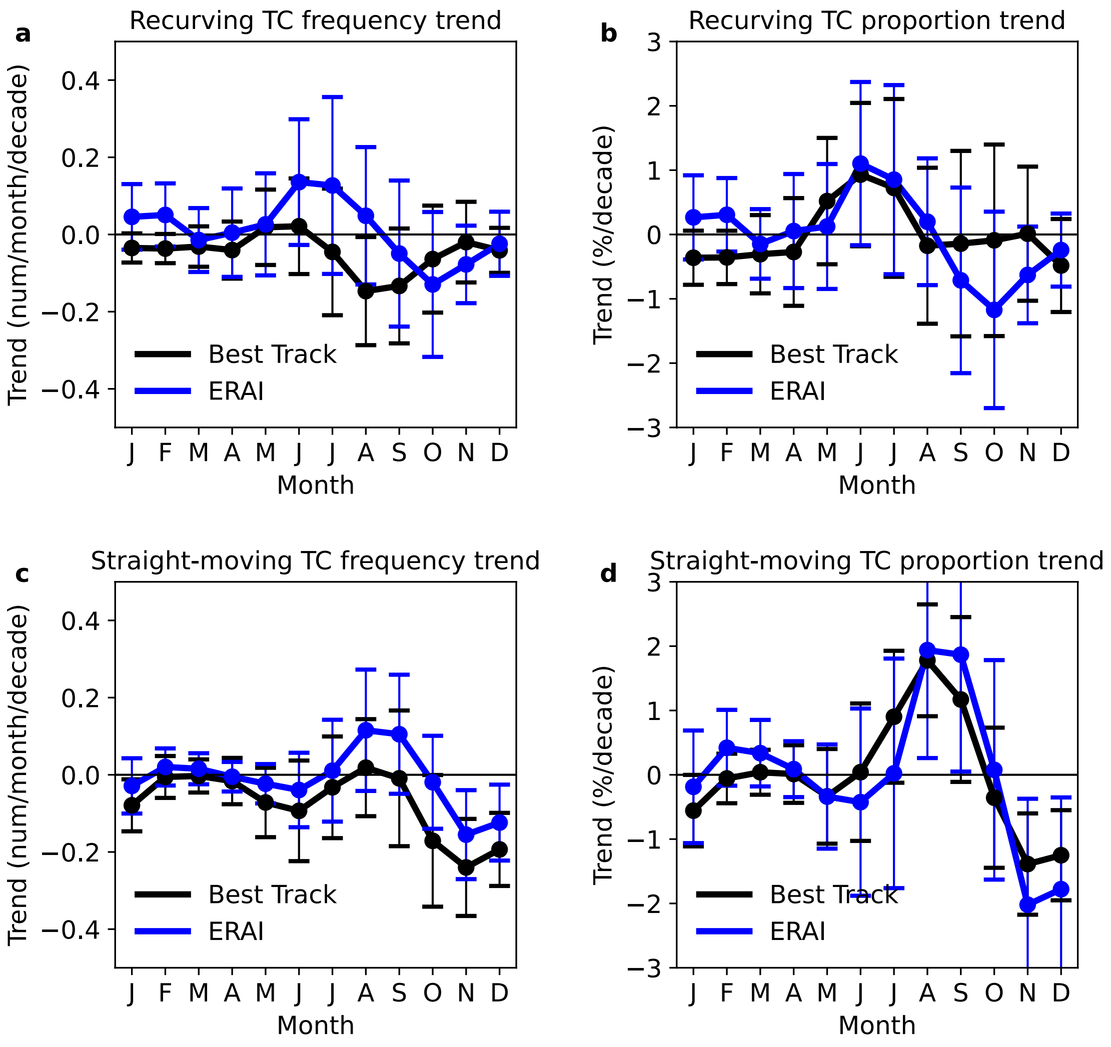

Supplementary Figure 8: Changes in seasonality of genesis for recurving and straight-moving tropical cyclones.

(a-b): Linear trends in 3-month rolling averages of monthly frequency and monthly relative frequency for recurving TCs, over 1979–2018, in the Best Track ensemble mean (black) and ERA-Interim (blue) TC datasets. (c-d) as (a-b), but for straight-moving TCs. The error bars show 95% confidence interval for the trends; the El Niño Southern Oscillation and Pacific Decadal Oscillation effects are removed by a multivariate regression.

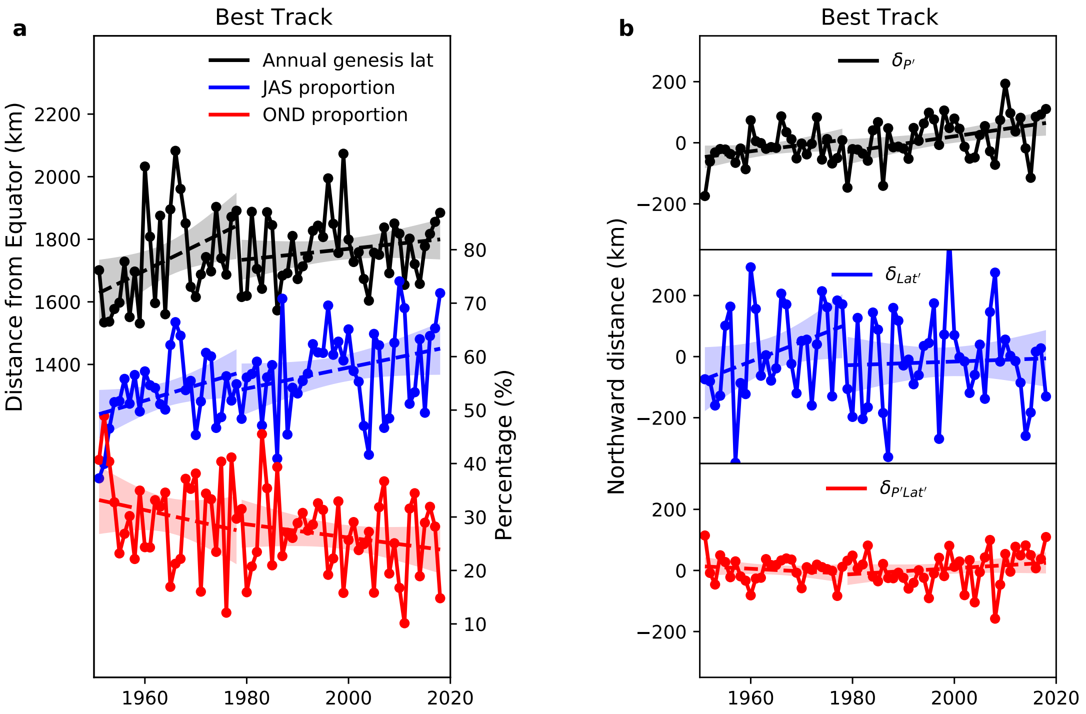

Supplementary Figure 9: Changes in annual-mean latitude of tropical cyclone genesis, and changes in three seasonality components, over the extended period 1951–2018.

(a) Timeseries of annual-mean latitude of TC genesis (black), and timeseries of peak-season (July–September; JAS, blue) and late-season (October–December; OND, red) relative frequencies of TC genesis in the year, in two epochs (the pre-satellite era 1951–1978 and the satellite era 1979–2018) of the whole period 1951–2018, in the Best Track ensemble mean. (b): Decompositions of annual-mean latitude of TC genesis by three seasonality contributing components, representing the effects of departures (from the respective monthly means) of monthly relative frequency ($\delta_{P'}$, black), monthly latitude ($\delta_{Lat'}$, blue) and the covariance of the two terms ($\delta_{P'Lat'}$, red), respectively, in two epochs of the whole period 1951–2018, in the Best Track ensemble mean. Dashed lines represent the linear trend, with shading showing 95% confidence interval for the linear fit; the El Niño Southern Oscillation and Pacific Decadal Oscillation effects are removed by a multivariate regression; latitude is converted to distance from the equator (km).

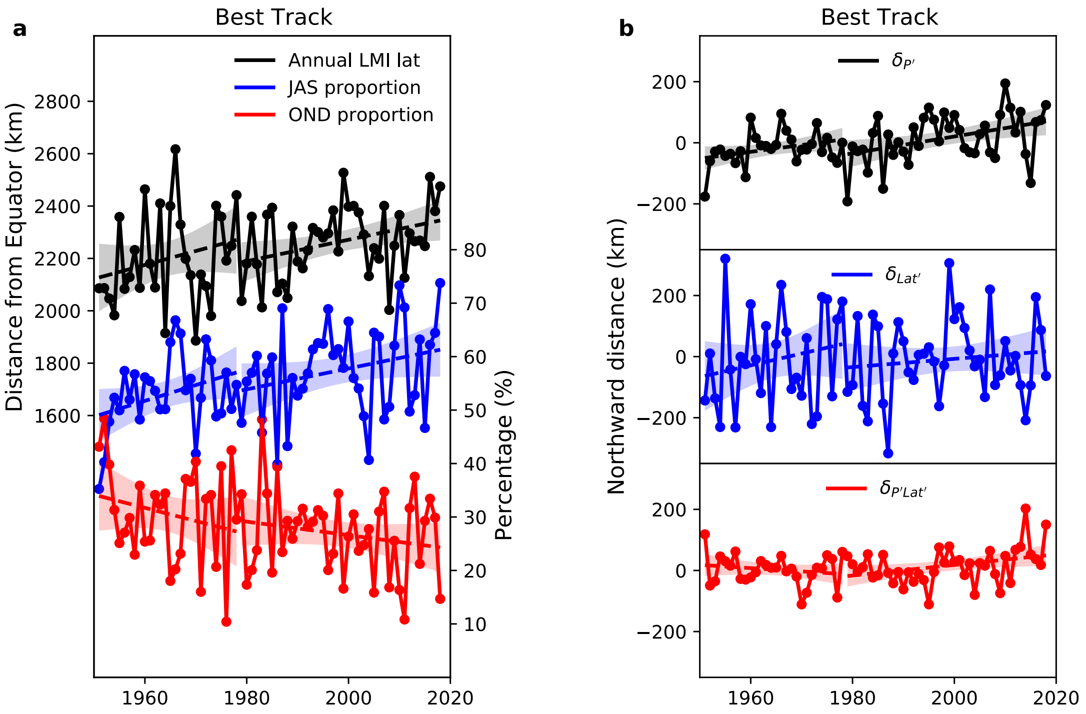

Supplementary Figure 10: Changes in annual-mean latitude of tropical cyclone lifetime-maximum intensity, and changes in three seasonality components, over the extended period 1951–2018.

As Supplementary Figure 9, but for TC lifetime-maximum intensity (LMI).

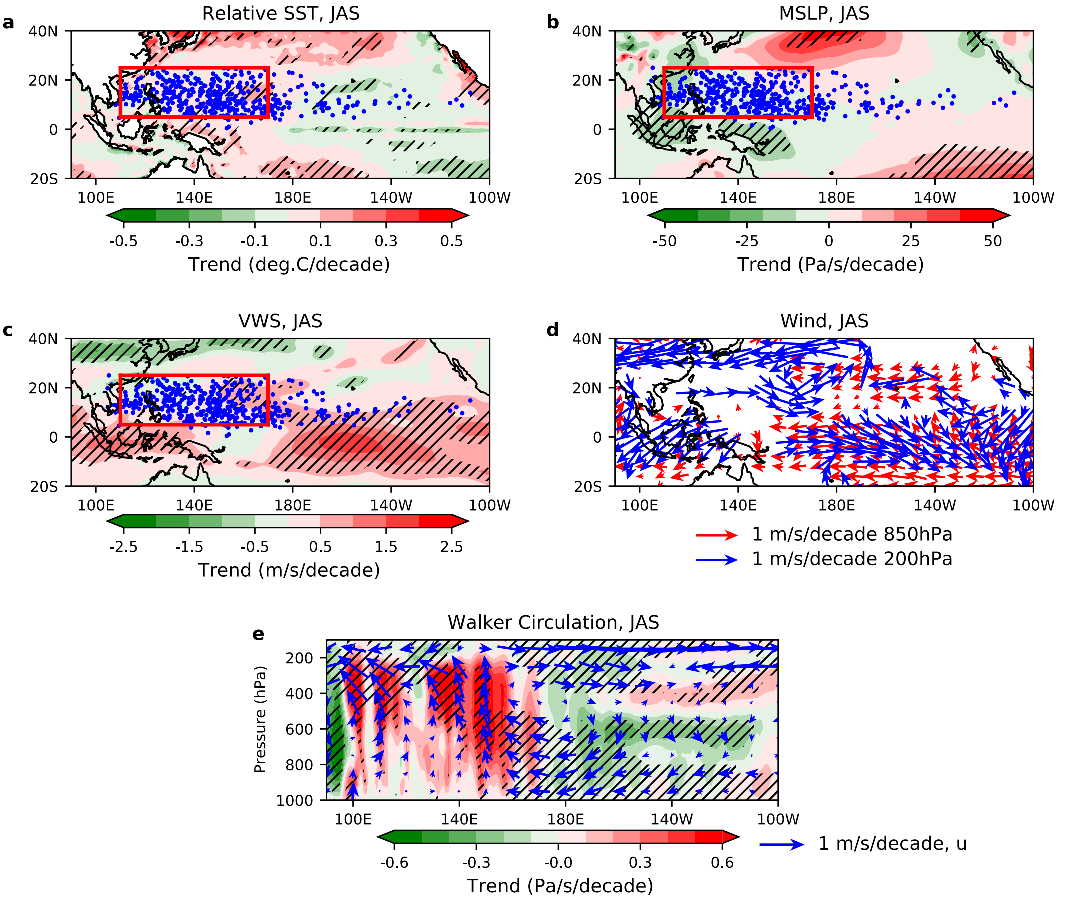

Supplementary Figure 11: Changes in peak-season environmental conditions in the Pacific. (a-d) Linear trends (shading) of relative sea surface temperatures (relative SSTs), mean sea level pressure (MSLP), vertical wind shear (VWS), and wind velocity at 850-hPa and 200-hPa (vectors), for the peak season (July–September; JAS), over 1979–2018, in ERA-Interim. Relative SSTs are SSTs minus the average of global SSTs in 30°S–30°N. Blue dots present peak-season TC genesis in the ERA-Interim TC dataset; red box (110–170°E and 5–25°N) represents the main development region (MDR) of peak-season TCs. (e) Linear trends of the peak-season Walker Circulation (vectors) and vertical velocity (shading; positive values for uplifting) in the zonal-vertical section averaged over 5°S–5°N, over 1979–2018, in ERA-Interim. Hatched areas in (a-c, e) and vectors in (d) show 95% confidence for the linear fit; the El Niño Southern Oscillation and Pacific Decadal Oscillation effects are removed by a multivariate regression; in (e), trends of vertical velocity are multiplied by 10^2 for display.

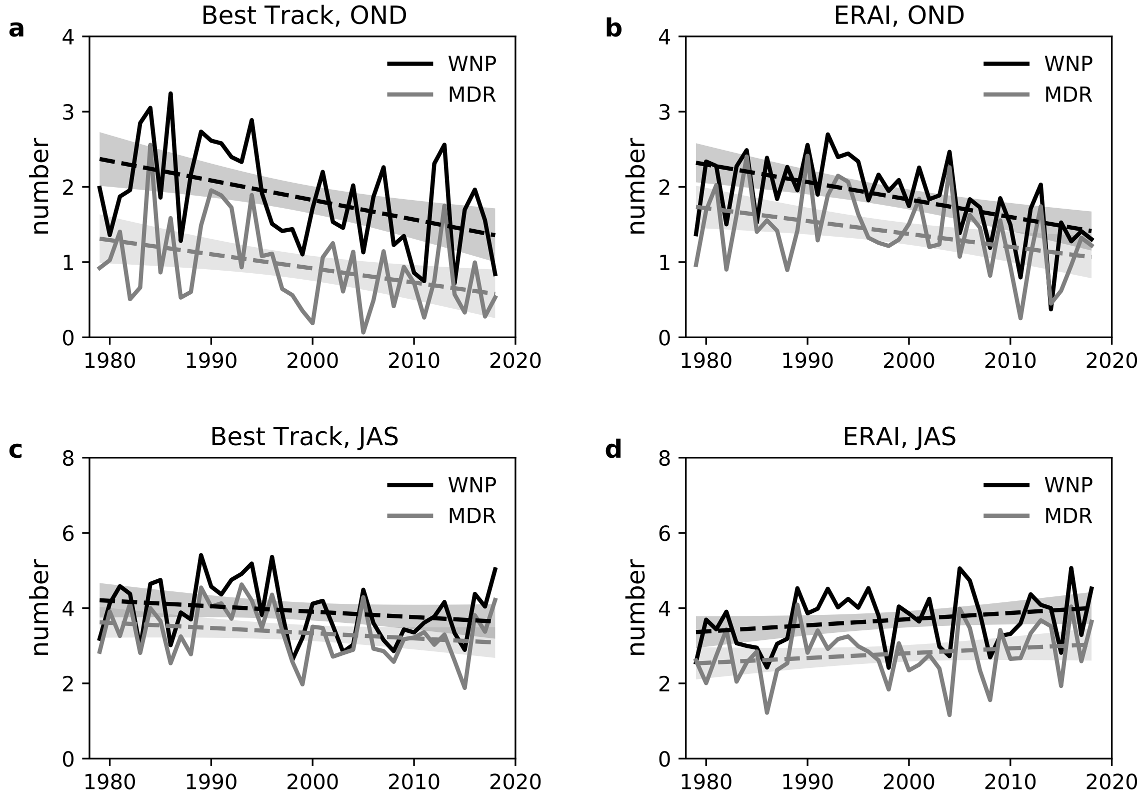

Supplementary Figure 12: Number of tropical cyclones formed in the main development region. (a-b) Timeseries of monthly frequency of western North Pacific TC genesis (black), and timeseries of monthly frequency of TC genesis developed in the main development region (MDR, grey), for the late season (October–December; OND), over 1979–2018, in the Best Track ensemble mean and ERA-Interim datasets. (c-d) as (a-b), but for the peak season (July–September; JAS). Dashed lines represent the linear trend, with shading showing 95% confidence interval for the linear fit; the El Niño Southern Oscillation and Pacific Decadal Oscillation effects are removed by a multivariate regression.

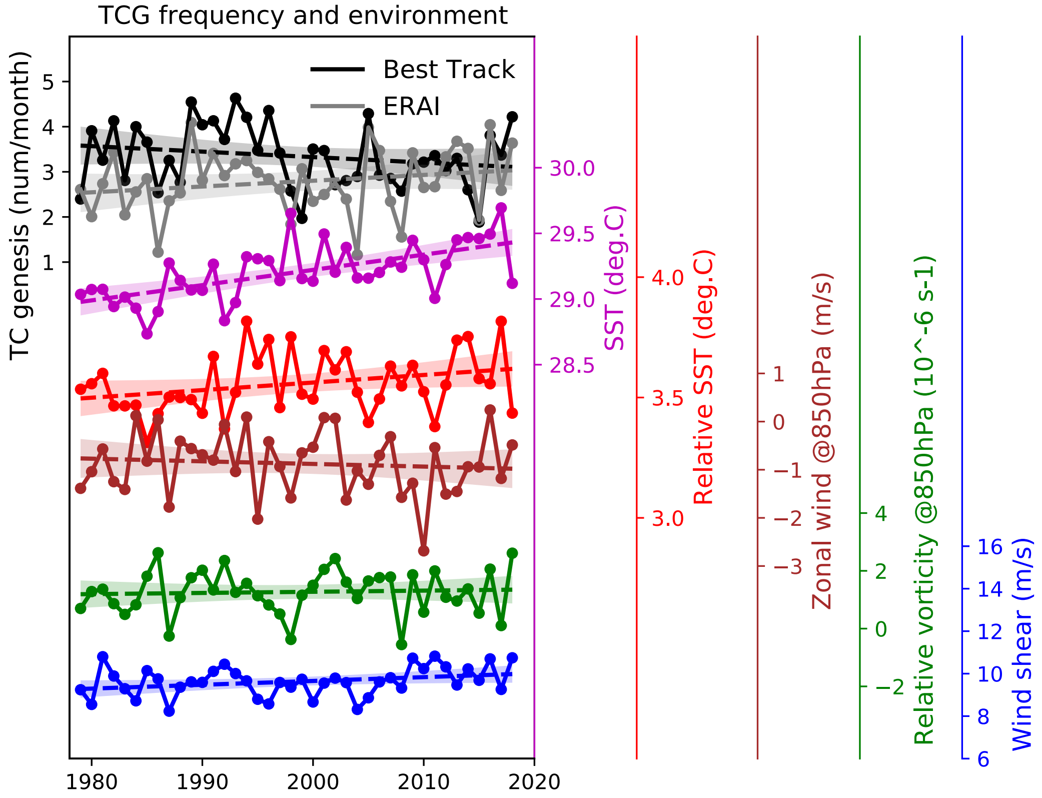

Supplementary Figure 13: Changes in peak-season tropical cyclone frequency and environmental conditions in the main development region.

Timeseries of monthly frequency of TC genesis (TCG) developed in the main development region (MDR, red box in Supplementary Figure 11), for the peak season (July–September; JAS), over 1979–2018, in the Best Track ensemble mean (black) and ERA-Interim (grey) TC datasets. Timeseries of sea surface temperatures (SSTs, magenta), relative SSTs (red), 850-hPa zonal wind (brown), 850-hPa relative vorticity (green) and vertical wind shear (blue), averaged over the MDR for the peak season over 1979–2018, in ERA-Interim. Relative SSTs are SSTs minus the average of global SSTs in 30°S–30°N. Dashed lines represent the linear trend, with shading showing 95% confidence interval for the linear fit; the El Niño Southern Oscillation and Pacific Decadal Oscillation effects are removed by a multivariate regression.

Supplementary Table 1: Linear trends in annual-mean latitude of tropical cyclones and seasonality components, in three Best Track sources.

Linear trends (km/decade) in annual-mean latitude and its three seasonality contributing terms ($\delta_{P'}$, $\delta_{Lat'}$ and $\delta_{P'Lat'}$) for western North Pacific TC track metrics, in the Best Track ensemble members, over two epochs (satellite and pre-satellite eras) of the whole period 1951–2018. The ensemble consists of Best Track data from the Joint Typhoon Warning Centre (JTWC), Japan Meteorological Agency (JMA) and China Meteorological Administration (CMA); as JMA Best Track data usually lack intensity records over the pre-satellite era, over this period the ensemble for lifetime-maximum intensity (LMI) and other related metrics (transit distances of developing and dissipating phases) only consists of JTWC and CMA data. The El Niño Southern Oscillation and Pacific Decadal Oscillation effects have been removed with a multivariate regression before estimating the trend; ± represents the 95% confidence interval of trend value, with an asterisk indicating significant trend at the 95% confidence; values with two asterisks indicate significant trend at the 90% confidence.

1979–2018	All-track-points (JTWC, JMA, CMA)	Genesis (JTWC, JMA, CMA)	LMI (JTWC, JMA, CMA)	Lysis (JTWC, JMA, CMA)
Total	53±35*, 86±38*, 79±32*	37±30*, 38±29*, 45±33*	60±36*, 66±29*, 56±35*	73±61*, 113±74*, 109±57*
$\delta_{P'}$	32±20*, 48±20*, 45±20*	20±17*, 24±16*, 25±18*	27±17*, 29±17*, 29±19*	43±26*, 52±28*, 52±31*
$\delta_{Lat'}$	7±30, 23±35, 23±27	6±26, 1±30, 11±28	18±29, 22±29, 12±29	7±55, 29±68, 29±56
$\delta_{P'Lat'}$	13±14**, 15±21, 11±14	10±14, 12±14**, 10±13	15±13*, 15±16**, 15±14*	23±20*, 32±26*, 28±21*
1979–2018	All-track-point transit distance (JTWC, JMA, CMA)	Transit distance (JTWC, JMA, CMA)	Developing phase transit distance (JTWC, JMA, CMA)	Dissipating phase transit distance (JTWC, JMA, CMA)
Total	9±25, 42±35*, 20±23**	36±57, 75±71*, 63±51*	24±26**, 28±24*, 11±21	13±52, 47±66, 52±42*
$\delta_{P'}$	11±6*, 21±10*, 14±7*	23±14*, 27±18*, 27±18*	7±4*, 4±3*, 4±3*	15±11*, 23±16*, 23±15*
$\delta_{Lat'}$	-5±22, 17±33, 3±26	1±50, 28±62, 18±55	11±24, 24±24, 1±19	-11±44, 7±58, 16±46
$\delta_{P'Lat'}$	3±13, 4±14, 3±12	13±25, 20±23**, 18±23	5±7, 3±8, 5±8	8±21, 17±21, 13±21
1951–1978	All-track-points (JTWC, JMA, CMA)	Genesis (JTWC, JMA, CMA)	LMI (JTWC, CMA)	Lysis (JTWC, JMA, CMA)
Total	46±85, -11±94, -23±94	98±65*, 46±66, 92±72*	51±79, 50±86	-44±167, -17±152, -89±152
$\delta_{P'}$	30±36**, 31±38, 40±35*	15±26, 16±24, 29±22*	11±26, 30±23*	25±37, 27±45, 47±42*

$\delta_{Lat'}$	18±73, -32±74, -43±70	84±53*, 41±52, 70±57*	42±69, 26±74	-49±165, -20±143, -120±129**
$\delta_{P'Lat'}$	-2±26, -11±31, -19±29	-1±21, -11±19, -7±19	-3±21, -6±22	-21±54, -24±53, -16±51
1951–1978	All-track-point transit distance (JTWC, JMA, CMA)	Transit distance (JTWC, JMA, CMA)	Developing phase transit distance (JTWC, CMA)	Dissipating phase transit distance (JTWC, CMA)
Total	-76±83**, -93±84*, -123±69*	-142±163**, -62±142, -180±134*	-47±55**, -42±50	-95±131, 139±101*
$\delta_{P'}$	4±10, 10±18, 9±17	10±21, 12±28, 18±29	-4±4**, 1±4	14±19, 18±27
$\delta_{Lat'}$	-64±75**, -82±78*, -114±66*	-132±165, -61±140, -190±126*	-41±54, -44±47**	-91±135, -146±92*
$\delta_{P'Lat'}$	-16±22, -22±27, -19±27	-20±45, -13±46, -9±46	-2±16, 1±15	-18±42, -11±38

Supplementary Table 2: Linear trends in annual-mean latitude of recurving and straight-moving tropical cyclones, in the Best Track ensemble mean.

Linear trends (km/decade) in annual-mean latitude and its three seasonality contributing terms ($\delta_{P'}$, $\delta_{Lat'}$ and $\delta_{P'Lat'}$) for recurving TCs and straight-moving TCs in the western North Pacific, in the Best Track ensemble mean, over the satellite era (1979–2018). The El Niño Southern Oscillation and Pacific Decadal Oscillation effects have been removed with a multivariate regression before estimating the trend; ± represents the 95% confidence interval of trend value, with an asterisk indicating significant trend at the 95% confidence; values with two asterisks indicate significant trend at the 90% confidence.

1979–2018	All-track-points (recurving, straight-moving)	Genesis (recurving, straight-moving)	LMI (recurving, straight-moving)	Lysis (recurving, straight-moving)
Total	68±45*, 74±47*	35±37**, 43±38*	47±45*, 74±37*	97±93*, 105±78*
$\delta_{P'}$	27±34, 52±25*	12±30, 33±18*	14±32, 37±20*	42±52, 54±31*
$\delta_{Lat'}$	19±31, 12±37	-3±33, 2±29	9±31, 28±30**	51±60, 39±75
$\delta_{P'Lat'}$	21±37, 11±19	25±29**, 9±20	24±29, 8±22	4±39, 11±28
1979–2018	All-track-point transit distance (recurving, straight-moving)	Transit distance (recurving, straight-moving)	Developing phase transit distance (recurving, straight-moving)	Dissipating phase transit distance (recurving, straight-moving)
Total	29±36, 21±31	63±84, 61±71**	12±29, 30±24*	50±79, 31±63
$\delta_{P'}$	18±13*, 15±8*	30±26*, 22±19*	2±7, 4±4*	28±24*, 17±16*
$\delta_{Lat'}$	23±30, 7±31	54±73, 37±76	12±34, 27±26**	42±58, 11±64
$\delta_{P'Lat'}$	-11±22, -1±15	-21±47, 3±31	-1±17, -1±12	-20±41, 3±28

Supplementary Table 3: Linear trends in annual-mean latitude of recurving and straight-moving tropical cyclones, in ERA-Interim TC dataset. Linear trends (km/decade) in annual-mean latitude and its three seasonality contributing terms ($\delta_{P'}$, $\delta_{Lat'}$ and $\delta_{P'Lat'}$) for recurving TCs and straight-moving TCs in the western North Pacific, in the ERA-Interim TC dataset, over the satellite era (1979–2018). The El Niño Southern Oscillation and Pacific Decadal Oscillation effects have been removed with a multivariate regression before estimating the trend; ± represents the 95% confidence interval of trend value, with an asterisk indicating significant trend at the 95% confidence; values with two asterisks indicate significant trend at the 90% confidence.

1979–2018	All-track-points (recurving, straight-moving)	Genesis (recurving, straight-moving)	LMI (recurving, straight-moving)	Lysis (recurving, straight-moving)
Total	85±59*, 84±63*	47±43*, 37±52	54±38*, 64±59*	93±134, 164±149*
$\delta_{P'}$	27±30**, 48±47*	5±18, 30±24*	3±21, 46±36*	25±46, 87±81*
$\delta_{Lat'}$	27±48, 30±36	31±37, 10±27	49±35*, 5±48	41±120, 31±104
$\delta_{P'Lat'}$	31±32**, 16±27	10±24, -4±23	2±21, 14±23	27±73, 45±68
1979–2018	All-track-point transit distance (recurving, straight-moving)	Transit distance (recurving, straight-moving)	Developing phase transit distance (recurving, straight-moving)	Dissipating phase transit distance (recurving, straight-moving)
Total	31±65, 49±59	46±146, 127±159	7±59, 27±74	39±110, 100±113**
$\delta_{P'}$	20±20*, 26±26*	19±36, 57±62**	-2±8, 15±16**	22±33, 42±49**
$\delta_{Lat'}$	-9±52, 11±45	10±124, 21±118	18±56, -5±65	-8±98, 26±74
$\delta_{P'Lat'}$	20±32, 13±32	17±79, 49±78	-8±30, 17±32	25±67, 32±56
